# Supplementary material for: High Performance Size Exclusion Chromatography-Inductively Coupled Plasma-Mass Spectrometry to Study the Copper and Cadmium Complexation with Humic Acids
Source: Molecules. 2019 Sep 3;24(17):3201. doi: 10.3390/molecules24173201 (PMC6749506; doi:10.3390/molecules24173201)
Supplement: Supplementary file 1 [file molecules-24-03201-s001.pdf]

Supplemetary materials

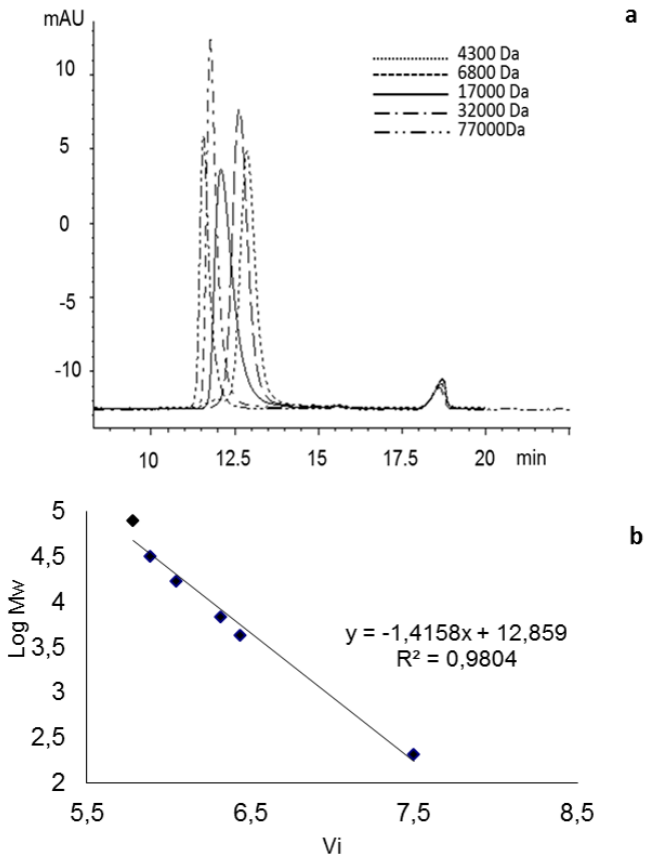

Figure S1. Chromatograms for the column calibration by molecular mass standards of polystyrene sulfonates and calibration curve.

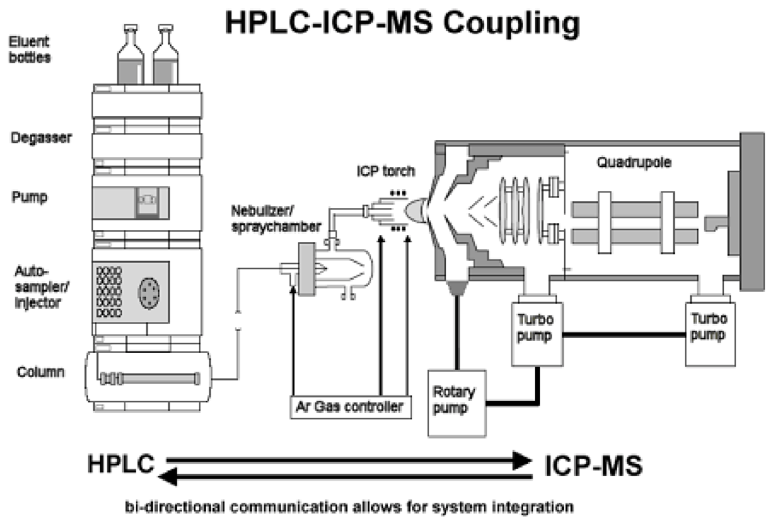

Figure S2. Scheme of the HPLC-ICP-MS

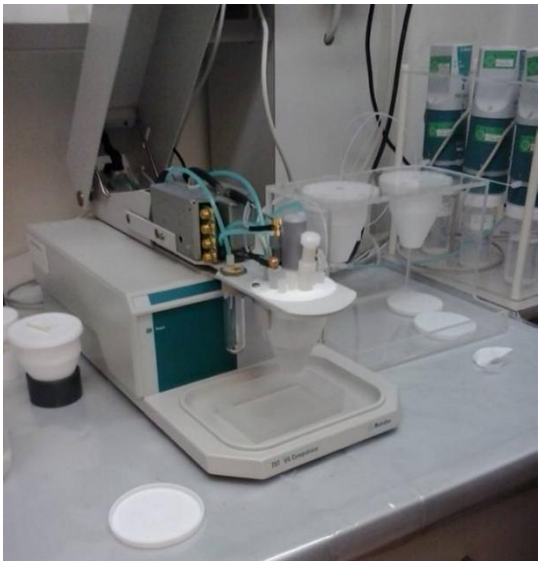

Figure S3. Photo of the Voltammetric instrumentation used for ASV measurements.

Table S1. Instrumental parameters of ICP-MS.

**Tuning Parameters**	**Value**	**Tuning Parameters**	**Value**
RF Power	1500 W	Extract 1	0 V
RF Matching	1.73 V	Extract 2	–170 V
Smpl Depth	8 mm	Omega Bias-ce	–30 V
Torch-H	0.5 mm	Omega Lens-ce	–0.4 V
Torch-V	–0.3 mm	Cell Entrance	–30 V
Carrier Gas	1.13 L/m	QP Focus	2V
Makeup Gas	0 L/m	Cell Exit	–40 V
Optional Gas	0%	OctP RP	170 V
Nebulizer Pump	0.1 rps	OctP Bias	–4 V
S/C temp	2 degC	QP Bias	–3 V
